# Plasma cell morphology as a trigger for the diagnosis of adult Fanconi syndrome

**DOI:** 10.1002/jha2.363

**Published:** 2021-12-20

**Authors:** Ibtisam Abdullah, Johan Niemann

**Affiliations:** ^1^ Division of Haematological Pathology Department of Pathology Northland District Health Board Northland New Zealand

A 76‐year‐old female presented with history of monoclonal gammopathy of undetermined significance (MGUS). Renal biopsy showed numerous rhomboid crystals within the tubular epithelium, prompting a diagnosis of light chain Fanconi syndrome. Bone marrow biopsy revealed 10% clonal plasma cells that displayed intracytoplasmic rhomboid crystalline inclusions, fine azurophilic granules (Figure 1A–C; bone marrow aspirate; Wright–Giemsa stain; 100× objective), and Auer rod‐like inclusions (Figure 1B,D, arrows). Additionally, crystal‐storing histiocytes were observed (Figure 1D,E; bone marrow aspirate; Wright–Giemsa stain; 100× objective). Adult Fanconi syndrome is a rare acquired renal condition affecting proximal renal tubules and is characterized by wasting of amino acids, phosphates, and various ions. Light chain Fanconi syndrome is slowly progressive monoclonal gammopathy characterized by monoclonal kappa light chain crystalline inclusions within the proximal tubular cells, bone marrow plasma cells, and sometimes bone marrow macrophages. The presence of Auer rod‐like inclusions within plasma cells, crystalline inclusions within bone marrow plasma cells and histiocytes in conjunction with renal impairment should trigger further investigations for associated adult Fanconi syndrome.

**FIGURE 1:**
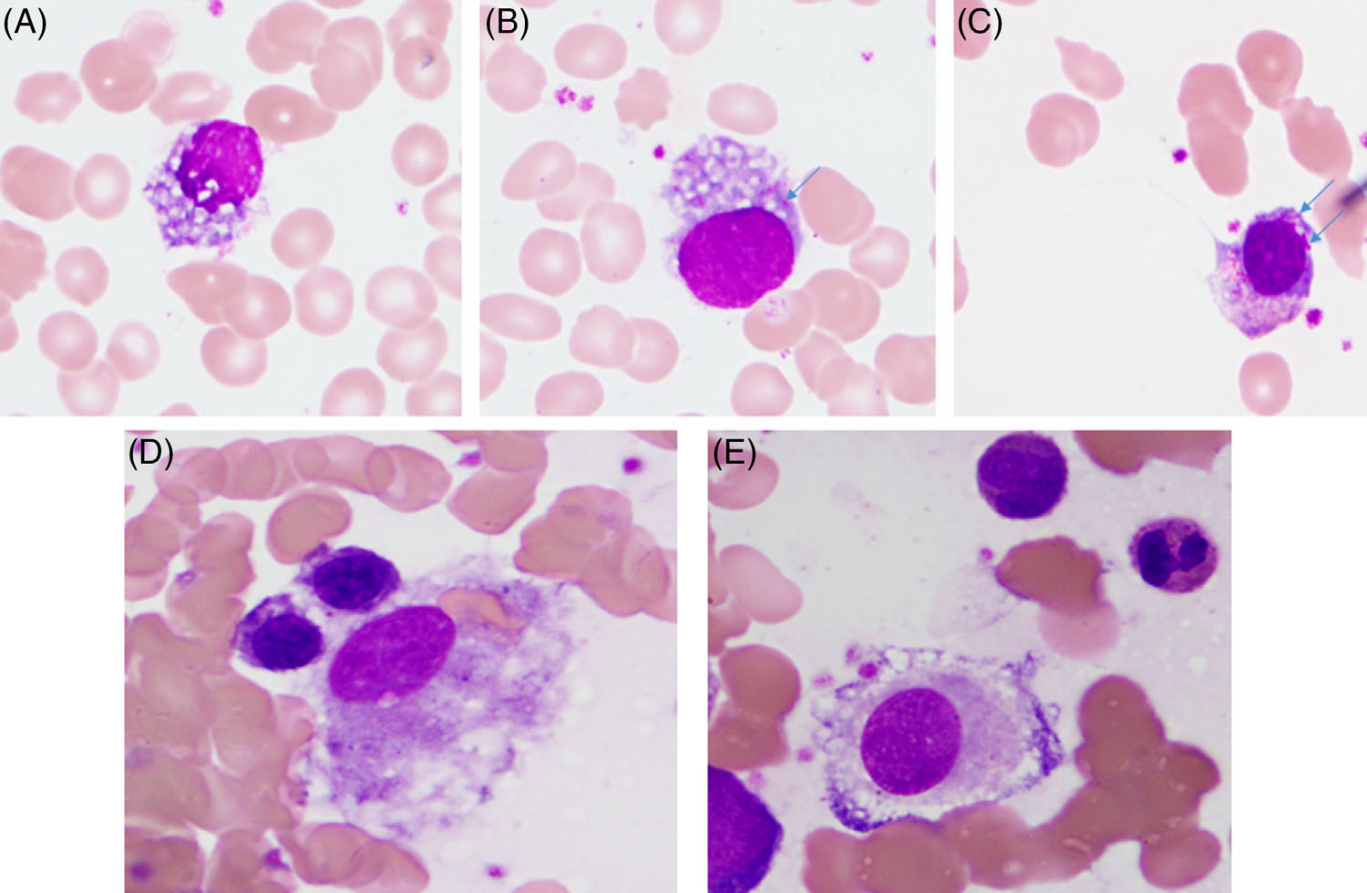
Micrograph from a bone marrow aspirate showing plasma cells with intracytoplasmic rhomboid crystalline inclusions (A–C) fine azurophilic granules (A–C), Auer rod‐like inclusions (B–C; arrows), and crystal‐storing histiocytes (D–E)

## CONFLICT OF INTEREST

The authors declare no conflict of interest.

## CONSENT STATEMENT

Written informed consent was obtained from the patient.

